# Detection of Dystrophin Dp71 in Human Skeletal Muscle Using an Automated Capillary Western Assay System

**DOI:** 10.3390/ijms19061546

**Published:** 2018-05-23

**Authors:** Tatsuya Kawaguchi, Emma Tabe Eko Niba, Abdul Qawee Mahyoob Rani, Yoshiyuki Onishi, Makoto Koizumi, Hiroyuki Awano, Masaaki Matsumoto, Masashi Nagai, Shinobu Yoshida, Sachiko Sakakibara, Naoyuki Maeda, Osamu Sato, Hisahide Nishio, Masafumi Matsuo

**Affiliations:** 1 Discovery Science and Technology Department, Daiichi Sankyo RD Novare Co., Ltd., Edogawa, Tokyo 134-8634, Japan; kawaguchi.tatsuya.sg@rdn.daiichisankyo.co.jp (T.K.); yoshida.Shinobu.z3@rdn.daiichisankyo.co.jp (S.Y.); 2 Department of Physical Therapy, Faculty of Rehabilitation, Kobe Gakuin University, Nishi, Kobe 651-2180, Japan; niba@med.kobe-u.ac.jp (E.T.E.N.); rani@reha.kobegakuin.ac.jp (A.Q.M.R.); 3 Department of Community Medicine and Social Healthcare Sciences, Kobe University Graduate School of Medicine, Chuo, Kobe 650-0017, Japan; nishio@med.kobe-u.ac.jp; 4 Modality Research Laboratories, Biologics Division, Daiichi Sankyo Co., Ltd., Shinagawa, Tokyo 140-8710, Japan; onishi.yoshiyuki.a2@daiichisankyo.co.jp (Y.O.); koizumi.makoto.h7@daiichisankyo.co.jp (M.K.); 5 Department of Pediatrics, Kobe University Graduate School of Medicine, Chuo, Kobe 650-0017, Japan; awahiro@med.kobe-u.ac.jp (H.A.); mmatsu@med.kobe-u.ac.jp (M.M.); natsu@med.kobe-u.ac.jp (M.N.); 6 Biomarker Department, Oncology Function, Daiichi Sankyo Co., Ltd., Shinagawa, Tokyo 140-8710, Japan; sakakibara.sachiko.bk@daiichisankyo.co.jp (S.S.); maeda.naoyuki.ct@daiichisankyo.co.jp (N.M.); 7 R&D Planning & Management Department, Daiichi Sankyo Co., Ltd., Shinagawa, Tokyo 140-8710, Japan; sato.osamu.ua@daiichisankyo.co.jp

**Keywords:** dystrophin, Dp71, Dp427, Simple Western, Western blotting, skeletal muscle

## Abstract

Background: Dystrophin Dp71 is one of the isoforms produced by the *DMD* gene which is mutated in patients with Duchenne muscular dystrophy (DMD). Although Dp71 is expressed ubiquitously, it has not been detected in normal skeletal muscle. This study was performed to assess the expression of Dp71 in human skeletal muscle. Methods: Human skeletal muscle RNA and tissues were obtained commercially. Mouse skeletal muscle was obtained from normal and DMD^mdx^ mice. Dp71 mRNA and protein were determined by reverse-transcription PCR and an automated capillary Western assay system, the Simple Western, respectively. Dp71 was over-expressed or suppressed using a plasmid expressing Dp71 or antisense oligonucleotide, respectively. Results: Full-length Dp71 cDNA was PCR amplified as a single product from human skeletal muscle RNA. A ca. 70 kDa protein peak detected by the Simple Western was determined as Dp71 by over-expressing Dp71 in HEK293 cells, or suppressing Dp71 expression with antisense oligonucleotide in rhabdomyosarcoma cells. The Simple Western assay detected Dp71 in the skeletal muscles of both normal and DMD mice. In human skeletal muscle, Dp71 was also detected. The ratio of Dp71 to vinculin of human skeletal muscle samples varied widely, indicating various levels of Dp71 expression. Conclusions: Dp71 protein was detected in human skeletal muscle using a highly sensitive capillary Western blotting system.

## 1. Introduction

The *DMD* gene is one of the largest human genes, consisting of 79 exons that span more than 2.4 Mb on chromosome X [1]. This gene produces a 14-kb transcript encoding dystrophin, a 427 kDa protein present at the subsarcolemma of skeletal muscle membranes. Dystrophin connects extra cellular matrix proteins with intra cellular actin by forming a dystrophin–dystroglycan complex, a scaffold for numerous signaling proteins [2]. The *DMD* gene encodes at least seven alternative promoters/first exons in introns, with transcription from each promoter producing a tissue-specific dystrophin isoform [1,3]. Recently, a novel development-specific promoter/first exon was found to produce a full-length transcript, providing further complexity in transcription [4]. Four promoters located in downstream introns produce shorter transcripts.

Dystrophin isoforms are named after their molecular weights: Dp427, Dp260, Dp140, Dp116 and Dp71. The full-length isoform, Dp427, is further classified by the tissue in which it is expressed. Dp427 expressed in skeletal muscle is called Dp427m, and its deficiency is the cause of Duchenne muscular dystrophy (DMD) (OMIM 310200), a fatal progressive wasting disease [3]. Dp71, the shortest dystrophin isoform, was cloned from human liver as non-muscle *DMD* gene product [5]. The Dp71 promoter/first exon (exon G1) is located in intron 62, such that exon G1 is spliced to exon 63, with the transcript containing all downstream exons (exon 63–79) [6,7]. Dp71 was found to be ubiquitously expressed, but not in skeletal muscle [5]. In addition, Western blotting failed to identify Dp71 in rat skeletal muscle [8]. Analysis of mouse myogenic cells by the Northern blot assay showed that Dp71 is expressed in myoblasts, is downregulated during in vitro myogenesis and is undetected in differentiated muscle cell cultures [6]. However, reverse transcription (RT)-PCR amplification identified Dp71 transcript in mouse skeletal muscle RNA [9]. It is understood that Dp71 is unexpressed in skeletal muscle [10].

Assays using animal-derived cell lines expressing Dp71 and Dp71-knockout mice found that Dp71 was involved in various cellular processes, including cell adhesion, water homeostasis, cell division, and nuclear architecture [10,11,12,13]. Despite these findings, fewer studies have assessed the function of human Dp71. Splicing variants of Dp71 were identified in human fetal neural tissue [14], and alternative splicing of Dp71 was shown to regulate nuclear or cytoplasmic localization in both HeLa and HEK293 human cell lines [15,16]. Moreover, Dp71 in the nucleus of HeLa cells was shown to form dystrophin–dystroglycan complexes [17].

Dp71 deficiency has been reported associated with non-muscular DMD phenotypes, such as severe cognitive impairment, retinal dysfunction, and short stature [18,19,20]. Dp71 may also act as a tumor suppressor [21], as Dp71-lamin complex were found to have tumor suppressive function in gastric cancers [22]. In contrast, knock-down of Dp71 reduced the malignancy of a lung adenocarcinoma cell line [23].

The important physiological roles of Dp71 suggested that this protein is expressed in human skeletal muscle. Here, the expression of Dp71 mRNA in human skeletal muscle was therefore assayed using RT-PCR amplification, and the expression of Dp71 protein was assayed using an automated capillary Western assay system, the Simple Western, which can precisely and accurately measure proteins at nanogram levels [24,25]. Moreover, Dp71 expression was assayed in skeletal muscles of both normal and DMD^mdx^ mice.

## 2. Results

### 2.1. Detection of Dp71 mRNA in Human Skeletal Muscle

**Figure 1:**
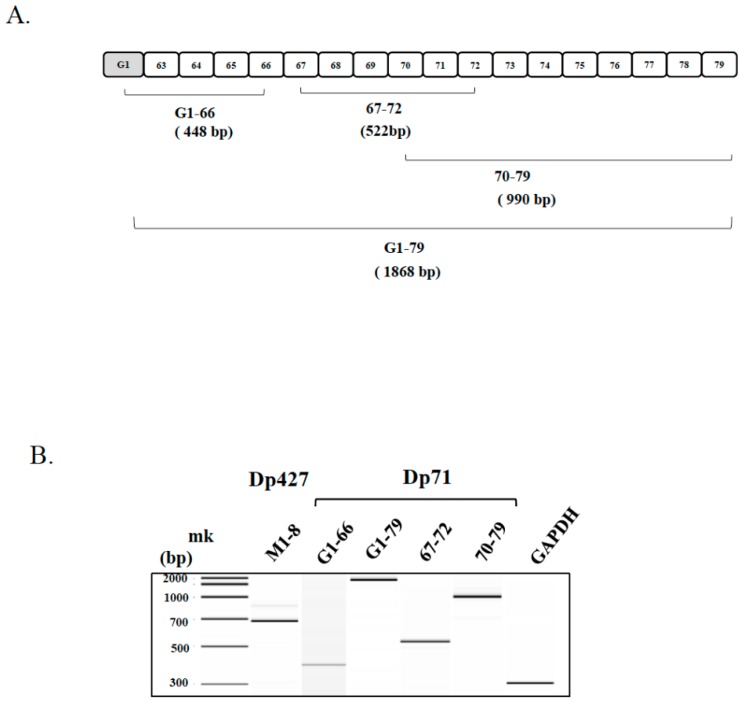
RT-PCR amplification of *DMD* transcript in human skeletal muscle. (**A**) Schematic description of Dp71 mRNA. The exon structure of Dp71 mRNA is shown. Boxes indicate exons. The shaded exon indicates Dp71-specific exon 1 (Exon G1). Numbers in boxes indicate exon number. Brackets and numbers under the bracket indicate the amplified fragment and exon number, respectively. Parenthesis indicates the size of the amplified fragment; (**B**) amplification of Dp427m and Dp71 mRNA: Dp427m and Dp71 mRNAs were RT-PCR amplified from human skeletal muscle total RNA. Electropherograms of amplified products are shown. The fragment extending from exon M1 to exon 8 was amplified as an expected size product (683 bp) (Dp427, M1-8). The fragment from exon G1 to exon 66 was also amplified as an expected size product (448 bp) (Dp71, G1–66). Full-length Dp71, from exon G1 to exon 79 was also amplified, yielding a single product of expected size (1868 bp) (Dp71, G1–79). Three fragments of Dp71, exon G1 to exon 66 (Dp71, G1–66); exon 67 to exon 72 (Dp71, 67–72), and exon 70 to exon 79 (Dp71, 70–79) were amplified and sequenced. As an internal control, the GAPDH cDNA was also amplified (GAPDH). Mk refers to size markers.

Ectopic human *DMD* transcript has been analyzed in lymphocytes by PCR amplification of 20 separate fragments of the full-length 14-kb long *DMD* cDNA [26,27]. This sensitive assay was applied to analyze Dp71 mRNA in human skeletal muscle (Figure 1A). Initially, the 5′ terminal fragment extending from exon M1 to exon 8 of Dp427m was RT-PCR amplified from human skeletal muscle RNA. This revealed a product (Figure 1B), indicating activation of Dp427m promoter. When the 5′ terminal fragment of Dp71, extending from exon G1 to exon 66 was RT-PCR amplified (Figure 1A), a single product with an expected size (448 bp) was obtained (Figure 1B). This indicated that Dp71 promoter is activated in human skeletal muscle.

To confirm the expression of Dp71 in human skeletal muscle, the full-length Dp71 cDNA, from exon G1 to exon 79, was PCR amplified. This amplification yielded a fragment of expected size (1868 bp) (Figure 1B). These findings indicated that full-length Dp71 mRNA was present in human skeletal muscle. Because Dp71 mRNA in other human tissues undergoes alternative splicing, involving the skipping of various exons, such as exons 71, 71–74, and 78 [10,16], the exon structure of the amplified product was examined by dividing the cDNA into three fragments, exon G1 to exon 66, exon 67 to exon 72, and exon 70 to exon 79, and amplifying each (Figure 1A). Sequencing of the three amplified products showed the presence of all exons, with no alternatively spliced exons. Dp71 mRNA in skeletal muscle was concluded to have all 18 exons intact.

### 2.2. Detection of Over-Expressed Dp71 Protein by the Simple Western

**Figure 2:**
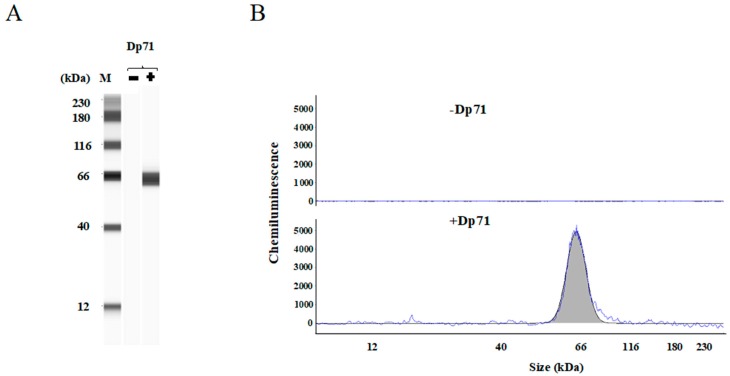
Detection of over-expressed Dp71 by the Simple Western. The Dp71 encoding plasmid was transfected into HEK293 cells and cell lysates were analyzed by the Simple Western using an antibody against the C-terminal of dystrophin. (**A**) Simple Western image pseudo-gel views of the molecular weight ladder and cell lysates are shown. A band near the 66 kDa molecular weight marker was identified in cells with the Dp71-expressing plasmid, but not in non-transfected cells; (**B**) electropherogram traces of cell lysates are shown. The Dp71 peak was not visible in non-transfected cells (−Dp71), whereas a band near the 66 kDa marker was observed in transfected HEK293 cells (+Dp71).

The above results suggested that Dp71 protein might be present in human skeletal muscle. To detect Dp71 protein in skeletal muscle, a highly sensitive protein measurement system, the Simple Western, was employed. The ability of the system to detect over-expressed Dp71 protein in HEK293 cells was initially assessed. A Dp71 encoding plasmid was constructed by cloning full length Dp71 cDNA into the pcDNA3 mammalian expression vector and transfected into HEK293 cells. The protein products, including over-expressed Dp71, were separated by the Simple Western. The result in pseudo-gel image showed a band close to the 66 kDa marker in cells transfected with the Dp71-encoding plasmid, but not in non-transfected cells (Figure 2A). Electropherogram traces showed a peak near the 66 kDa marker in transfected cells, whereas the peak was not visible in non-transfected cells (Figure 2B). These findings indicated that the Simple Western could detect Dp71 in cell lysates, localizing this protein near the 66 kDa marker.

### 2.3. Detection and Suppression of Dp71 in Rhabdomyosarcoma Cells

**Figure 3:**
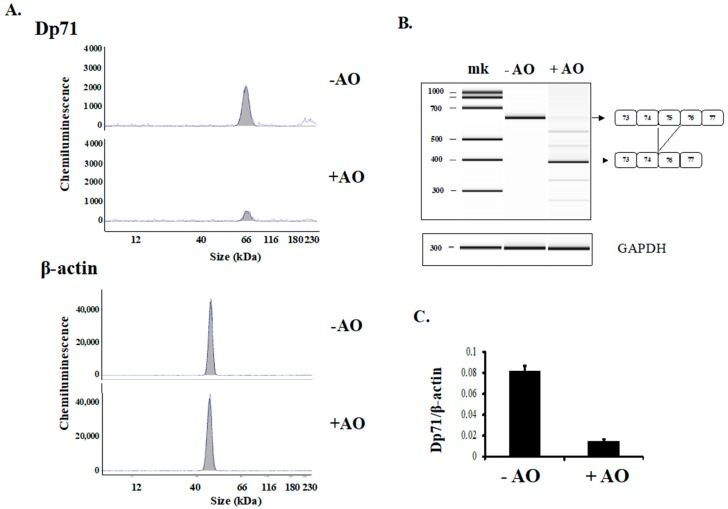
Detection of Dp71 in rhabdomyosarcoma cells. (**A**) Detection and suppression of Dp71 in CRL-2061 cells. CRL-2061 cell lysates were analyzed by the Simple Western. Electropherogram traces of the lysate are shown. A Dp71 peak was observed near the 66 kDa marker (−AO). The identity of this peak as Dp71 was confirmed by disrupting Dp71 mRNA reading frame by transfection of the antisense oligonucleotides AODys75-3. The transfected cells were harvested and analyzed. The Peak height of Dp71 was much lower (+AO), indicating that disruption of the Dp71 mRNA reading frame reduced Dp71 production. β-actin was used as the loading control (bottom panel); (**B**) effect of AO that induces DMD exon 75 skipping. AODys75-3 against the exonic splicing enhancer sequence within DMD exon 75 was synthesized and its ability to induce exon 75 skipping was assessed. RT-PCR products encompassing exons 73 to 77 are shown. In non-transfected CRL-2061 cells, normal product was obtained (−AO). In AODys75-3 transfected cells, the normal sized band was barely visible and the band appeared as a small size band having a deletion of exon 75 (+AO), indicating that AODys75-3 induced 75 skipping. The exon structure of the amplified product is shown schematically on the right; (**C**) Dp71/β-actin ratio in AO non-transfected and transfected cells. The Dp71/β-actin ratio was calculated in AO non-transfected and transfected cells in Panel A. Bars represent means ± S.E.M.

The Simple Western was utilized to detect Dp71 in the CRL-2061 rhabdomyosarcoma cell line, which had been utilized as a skeletal muscle surrogate [28]. Analysis of a CRL-2061 cell lysate showed a peak around 66 kDa in the electropherogram trace (Figure 3A). The identity of this peak as Dp71 was based on the identification of Dp71 overexpressed in HEK293 cell. To further confirm the identity of this peak, the expression of Dp71 in these cells was specifically suppressed by introducing an antisense oligonucleotide (AO) that disrupted the reading frame of Dp71 mRNA by inducing exon 75 skipping. AO mediated exon skipping has been used to change out-of-frame translation of *DMD* mRNA to in-frame translation, restoring dystrophin expression in DMD patients [29]. Conversely, AO-induced exon 75 skipping was employed to suppress Dp71 expression by changing the reading frame to out-of-frame, as exon 75 is an out-of-frame exon. The AO was designed to cover the exonic splicing enhancer sequence within the exon 75 sequence [30,31]. Accordingly, one AO (AODys75-3), consisting of ENA and 2’-*O*-methyl RNA, was synthesized and transfected into CRL-2061 cells. Thereafter, the Dp71 mRNA fragment extending from exon 73 to exon 77 was RT-PCR amplified. In un-transfected cells, the band corresponding to Dp71 mRNA was clearly visible (Figure 3B). In contrast, the band corresponding to the normal splicing product was barely visible in AODys75-3 transfected cells. Rather, a smaller size band was present (Figure 3B). Sequencing of the latter showed the absence of the exon 75 sequence, indicating that AODys75-3 induced exon 75 skipping.

Dp71 protein in AODys75-3 treated CRL-2061 cells was assessed using the Simple Western method. The Dp71 peak was lower in AODys75-3 treated than in untreated cells (Figure 3A), whereas the β-actin peak was at a similar level in both (Figure 3A). Calculation of the AUCs of Dp71 and β-actin peaks in treated and untreated cells showed that the mean Dp71/β-actin ratio was 0.015 in treated cells and found 0.083 in untreated cells, confirming that disruption of the Dp71 reading frame with AO reduced the Dp71 AUC. These findings indicated that the Dp71 peak identified by the Simple Western was the product of Dp71 mRNA and that the Simple Western was sufficiently sensitive to detect changes in Dp71 expression in a small amount of protein sample.

### 2.4. Detection of Dp71 Protein in Skeletal Muscles from Normal and DMD^mdx^ Mice

**Figure 4:**
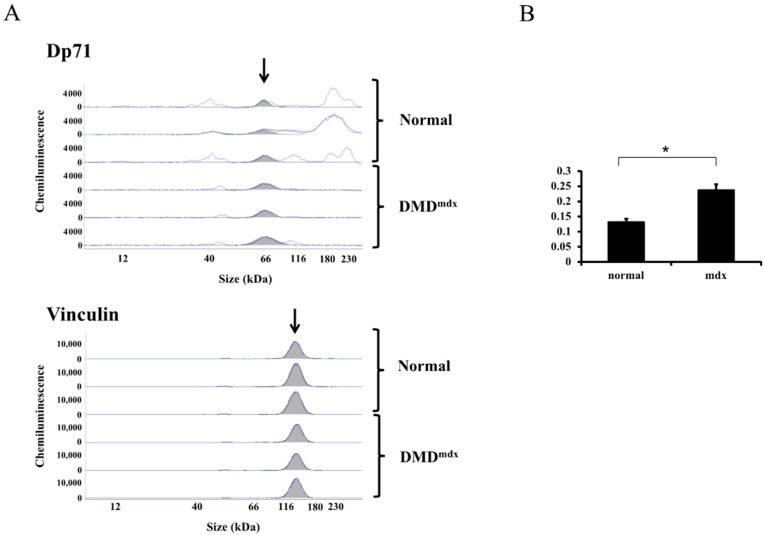
Dp71 in skeletal muscles from normal and mdx mice. (**A**) Skeletal muscles of C57BL/6 mice and DMD^mdx^ mice were analyzed three times by the Simple Western, with the results shown as electropherogram traces. The Dp71 peak recognized by anti-dystrophin antibody, ab15277, was present in all three samples from normal and DMD*^mdx^* mice (top). Simultaneously, vinculin was analyzed as a loading control in the same samples (bottom). The arrows indicate the Dp71 and vinculin peaks; (**B**) the ratio of Dp71 to vinculin in samples from normal and DMD*^mdx^* mice. Dp71 to vinculin ratio was calculated from the AUC of each peak. Bars represent means ± S.EM. * *p* < 0.05 compared with normal mice.

The Simple Western was also used to analyze Dp71 in skeletal muscles of both normal C57BL/6 mice and *mdx* (DMD^mdx^ ) mice, a dystrophin deficient model of DMD (Figure 4A). Assay of normal skeletal muscle showed a protein peak around 66 kDa, which, based on its mobility and reactivity to AO- mediated exon skipping, was designated Dp71. Interestingly, this Dp71 peak was also detected in DMD^mdx^ mice (Figure 4A). To compare Dp71 expression in these mice, their Dp71/vinculin ratios were calculated at three experiments by measuring the AUC of each peak. The mean ratio was significantly higher in DMD than in normal mice (0.24 vs. 0.13, *p* < 0.01; Figure 4B), indicating that Dp71 expression is high in DMD^mdx^ mice.

### 2.5. Detection of Dp71 in Human Skeletal Muscle

**Figure 5:**
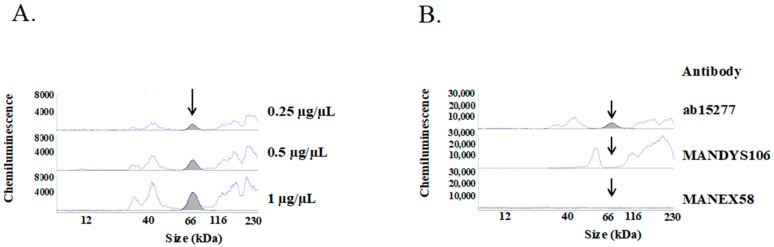
Dp71 in human skeletal muscle. (**A**) Dose-responsive changes in the Dp71 peak. Human skeletal muscle lysates (SK1) were gradient diluted and analyzed by the Simple Western using an antibody against the C-terminal domain of dystrophin, ab15277. A peak corresponding to Dp71 was identified near 66 kDa marker, with peak height decreasing as loading protein decreased (arrow); (**B**) Dp71 not recognized by antibodies to the dystrophin rod-domain. The specificity of the Dp71 peak was examined with several primary antibodies, with results shown as electropherogram traces of the Simple Western. The peak was recognized by antibody against the C-terminal domain of dystrophin (ab15277) (shaded peak), but not by antibodies against the rod domain of dystrophin, including antibodies against exon 43 (MANDYS106) and exon 58 (MANEX58). The arrows indicate the Dp71 peaks.

Simple Western analysis of Dp71 expression in normal human skeletal muscle showed a peak near the 66 kDa marker corresponding to Dp71 (Figure 5A). Increasing the loading dose of muscle lysates increased the height of the peak, indicating that peak height was dose dependent (Figure 5A). To confirm that this peak was a truncated form of dystrophin, the antibody used in the assay was replaced with antibodies against the rod domain of dystrophin (Figure 5B). The Dp71 peak recognized by the antibody to the C-terminal domain of dystrophin could not be detected by antibodies against amino acid sequences encoded by exon 43 (MANDYS106) and exon 58 (MANEX58). This finding indicated that the protein possesses the C-terminal domain but not the rod-domain of dystrophin, most of which is absent from Dp71. This confirmed that human skeletal muscle expresses Dp71.

**Figure 6:**
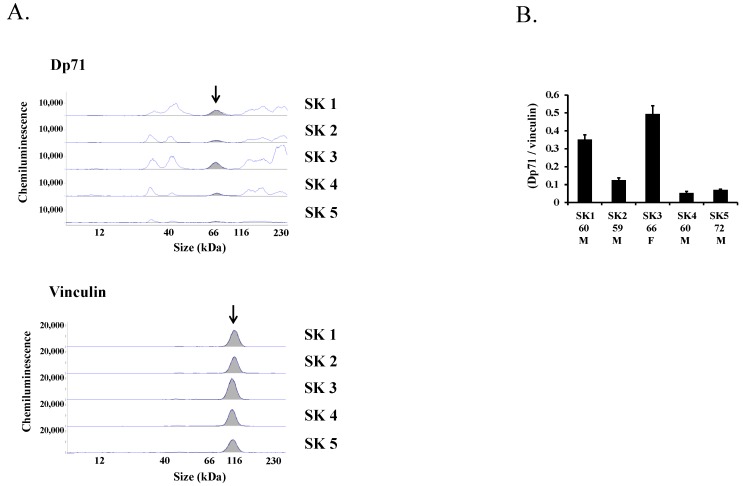
Levels of Dp71 protein expression in human skeletal muscles. Five samples of human skeletal muscles (SK1-SK5) were analyzed using the Simple Western system with antibody against the C-terminal domain of dystrophin (ab15277), and anti-vinculin antibody as a loading control. Electropherogram traces of are shown (**A**). The Dp71 peaks (arrows) in the samples were detected at the same location but at different heights. The Dp71/vinculin ratio (mean ± S.E.M.) was calculated in each sample (**B**) and found to range between 0.05 and 0.49. Numbers under the columns indicate sampling age (years). M and F indicate male and female, respectively.

Dp71 expression was also assayed in four additional samples of human skeletal muscle. The peak of Dp71 was present in electropherogram images of all five skeletal muscle samples, but their AUC differed (Figure 6A). Calculation of the Dp71/vinculin ratios in these samples showed that they ranged from 0.05 to 0.49 (Figure 6B), indicating a wide range of Dp71 expression level in normal human muscles.

## 3. Discussion

This report showed that dystrophin Dp71 mRNA and protein were present in human skeletal muscle and that Dp71 protein was present in skeletal muscles from normal and DMD mice, indicating that Dp71 is expressed in skeletal muscles across species. The expression of Dp71 in skeletal muscle was not unexpected, in as much as Dp71 mRNA has a house-keeping type promoter [32] and Dp71 is involved in the processes of cell cycle progression and cell differentiation [12,13]. Dp71 mRNA in human skeletal muscle consists of all exons, from exon G1 to exon 79, with no alternative exons. This was unexpected, as Dp71 mRNA has shown various alternative splicing patterns in other cells and tissues [10,16]. Similarly, Dp427m in skeletal muscle has not shown alternative splicing patterns in this region [1], suggesting that the splicing of Dp71 transcript in skeletal muscle is regulated by factors that regulate the splicing of Dp427m transcript.

This study also showed that Dp71 was expressed in a rhabdomyosarcoma cell line, CRL-2061 [28,33]. Because rhabdomyosarcoma is a muscle-derived sarcoma, CRL-2061 cells have been used experimentally as a skeletal muscle surrogate. The finding, that Dp71 was expressed in CRL-2061 cells, was consistent with Dp71 expression in skeletal muscle. However, the expression of Dp71 in this sarcoma cell line was incompatible with the hypothesis that Dp71 is a tumor suppressor [21]. Further studies are required to assess the roles of Dp71 in rhabdomyosarcoma carcinogenesis.

Dp71 protein was detected in skeletal muscle by a highly sensitive capillary Western system, the Simple Western. Although use of this system for protein analysis is expanding [24,25,34,35], it has not, to our knowledge, been utilized previously to detect dystrophin. Conventional Western blotting analysis is more limited, as its results are semi-quantitative and its productivity relatively low. The identity of the protein detected by the Simple Western as Dp71 was confirmed by its electrophoretic mobility and its reactions with anti-Dp71 antibody, as well as the suppression of its expression by an AO that disrupted the Dp71 reading frame. Further validation, however, is necessary to apply the Simple Western to the quantification of Dp427.

Our identification of Dp71 in skeletal muscle was in disagreement with studies finding that Dp71 was not expressed in skeletal muscle [10]. The inability to detect Dp71 in previous studies was likely due its low sensitivity of Northern and Western blotting assays designed to detect mRNA and protein, respectively. Our analysis, therefore, used highly sensitive methods, RT-PCR amplification and the Simple Western system, to detect Dp71 mRNA and protein, respectively.

Skeletal muscles of patients with DMD are frequently assayed immunohistochemically using an antibody against the C terminal domain of dystrophin. Because this antibody reacts with both Dp427 and Dp71, Dp71 should be identified in most DMD patients with *DMD* gene mutations located upstream of the Dp71 promoter [27]. This antibody, however, has failed to detect dystrophin in skeletal muscles of DMD patients [36,37] and in the skeletal muscles of DMD^mdx^ mice [35], despite our finding showing that Dp71 was expressed in the skeletal muscles of these mice. These findings suggested that an immunohistochemical method designed to detect Dp427 was unable to detect Dp71 due to its much lower level of expression in these tissues.

The localization of Dp71 in skeletal muscle remains unclear. Dp71 has been localized to various cellular fractions, including the plasma membrane, cytosol, nuclear membrane and nuclear matrix [10]. Certain subisoforms of Dp71 have been detected in the nuclear fraction [11,16]. In contrast, our study did not detect any specific subisoform of Dp71 in skeletal muscle; rather, we detected full-length Dp71, containing all exons from exon G1 to exon 79. It was difficult to determine Dp71 localization in skeletal muscle from its exon structure.

The level of Dp71 protein expression in human skeletal muscles was found to vary widely. Of the five samples tested, two had high and three had low Dp71/vinculin ratios, with the lowest level being nearly-one tenth that of the highest level. Differences in Dp71 level may be due to differences in muscle location; sampling method, such as biopsy or autopsy; or sex. The level of the transcriptional factor heat shock factor 1 is responsive to shock processes, including elevated temperature, oxidative stress, heavy metals, and bacterial and viral infection [38]. As Dp71 expression is controlled by the heat shock factor 1 [39], stress may alter Dp71/vinculin ratio. Moreover, Dp71 was recently shown to be regulated by phosphorylation and the ubiquitin-proteasome system in neuronal cells [40]. It may be necessary to measure multiple reference proteins for sample quality check. Further studies on the mechanism of Dp71 expression in skeletal muscle samples may reveal its regulatory system.

This study also used an AO against *DMD* exon 75 to disrupt the Dp71 reading frame, inducing exon 75 skipping. Clinically, this AO may restore the *DMD* reading frame in DMD patients [41]. In future, this AO can be used to treat DMD patients with deletions of *DMD* exons 69–74.

This study had several limitations. Importantly, although we assessed Dp71 in normal skeletal muscle samples, we did not analyze its expression in muscle samples from DMD patients. It is necessary therefore to analyze Dp71 expression in Dp427-deficient muscles.

Additionally, one article was published to detect Dp427 using the Simple Western during the process of reviewing of this article [42].

## 4. Materials and Methods

### 4.1. Cells

The HEK293 cell line was obtained from the Japanese Collection of Research Bioresources (JCRB) Cell Bank (Osaka, Japan) and cultured in Dulbecco’s modified Eagle’s medium (DMEM, Gibco/Life Technologies, Grand Island, NY, USA), supplemented with 10% fetal bovine serum (FBS, Moregate Biotech, Bulimba, Australia) and 1% antibiotic-antimycotic reagents (Gibco/Life Technologies). The SHSY-5Y and CRL-2061 cell lines were obtained from American Type Culture Collection (ATCC, Manassas, VA, USA). SHSY-5Y cells were cultured in a 1:1 mixture of DMEM and Ham’s F12 (Wako Pure Chemical Industries Ltd., Osaka, Japan), and CRL-2061 cells were cultured in RPMI medium (Gibco/Life Technologies). These were supplemented with 10% FBS (Gibco/Life Technologies) and 1% antibiotic-antimycotic reagents. All cell lines were cultured at 37 °C in a 5% CO_2_ humidified incubator. Prior to use, cultured cells were rinsed twice with phosphate-buffered saline (PBS, Sigma–Aldrich Co., St. Louis, MO, USA) and collected using a cell scraper.

### 4.2. Muscle Samples

Mouse gastrocnemius muscles were dissected from 5-week-old C57BL/6 mice and DMD^mdx^ mice, a mouse model of DMD obtained from the Central Institute for Experimental Animals (Kanagawa, Japan). Fresh frozen human skeletal muscle samples (SK1, SK2, SK3, SK4 and SK5) obtained at autopsy were purchased from Asterand Biosciences (Detroit, MI, USA).

### 4.3. mRNA Analysis

Total RNA from human skeletal muscle was obtained from a human total RNA Master Panel II (Clontech Laboratories, Inc., Mountain View, CA, USA). RNA was extracted from harvested SHSY-5Y, HEK293 and CRL-2061 cells using a High Pure RNA isolation kit (Roche Diagnostics, Basel, Switzerland). cDNA was synthesized from 0.5 µg of total RNA using random primers as described [43]. The *DMD* transcript corresponding to a fragment extending from exon M1 to 8 was PCR amplified as described [26,44]. The 5′ end of the Dp71 transcript was PCR amplified using a forward primer in exon G1 (ExG1ANf: 5′-TTGCAGCCATGAGGGAACAG-3′) and a reverse primer in exon 66 (c66r: 5′-GGACACGGATCCTCCCTGTTCG-3′). A full-length Dp71 transcript was PCR amplified using the forward primer in exon G1 (ExG1ANf) and a reverse primer in exon 79 (5F: 5′-ATCATCTGCCATGTGGAAAAG-3′). Full-length Dp71 was also amplified as three separate fragments, a 5′ fragment from exon G1 to exon 66, a central fragment from exon 67 to exon 72 and a 3′ fragment from exon 70 to exon 79. The central fragment was amplified using the primers 5E (5′-ATTGAGCCAAGTGTCCGG-3′) and c72r (5′-TATCATCGTGTGAAAGCTGAG-3′), and the 3′ fragment was amplified using the primers c70f (5′-CAGGAGAAGATGTTCGAGAC-3′) and 5F. For the exon 75 skipping experiment, a fragment extending from exons 73 to 77 was PCR amplified using a forward primer in exon 73 (Ex73Anf:5′-GCTAGCAGAAATGGAAAACAGCA-3′) and a reverse primer in exon 77 (Ex77Anr:5′-CACCTCCTCTAACCCTGTGC-3′). As a loading control, glyceraldehyde dehydrogenase (GAPDH) cDNA was PCR amplified [45].

All PCR amplifications were performed in a total volume of 10 µL, containing 1 µL of cDNA, 1 µL of 10× ExTaq buffer (Takara Bio, Inc., Shiga, Japan), 0.25 U of ExTaq polymerase (Takara Bio, Inc.), 500 nM of each primer, and 250 µM dNTPs (Takara Bio, Inc.), on a Mastercycler Gradient PCR machine (Eppendorf, Hamburg, Germany). The amplification protocol consisted of an initial denaturation at 94 °C for 5 min, followed by 30 cycles (18 for GAPDH) of denaturation at 94 °C for 0.5 min, annealing at 59 °C for 0.5 min, and extension at 72 °C for 1 min. PCR-amplified products were electrophoresed using a DNA 1000 LabChip kit on an Agilent 2100 Bioanalyzer (Agilent Technologies, Santa Clara, CA, USA). For large products, a DNA 7500 LabChip kit (Agilent Technologies) was used.

For sequencing, PCR-amplified products visualized by agarose gel electrophoresis were excised from the gel with a sharp razor blade, pooled, and purified using QIAquick gel extraction kits (QIAGEN, Inc., Hilden, Germany). The purified products were sequenced directly or subcloned into the pT7 blue T vector (Novagen, Inc., San Diego, CA, USA) for sequencing. Sequencing by the Sanger method was performed using a PreMix sequencing system (Greiner Bio-One Co., Ltd., Tokyo, Japan).

### 4.4. Over-Expression of Dp71

A Dp71-expressing plasmid was constructed by inserting the Dp71 coding sequence, consisting of *DMD* exons G1 and 63–79, into the plasmid pcDNA3, a mammalian expression vector with CMV promoter (Invitrogen, Thermo Fisher Scientific Inc., Carlsbad, CA, USA). This construct was synthesized by FASMAC Co., Ltd. (Kanagawa, Japan) and its sequence was confirmed by sequencing (data not shown but available on request). HEK293 cells grown to 80% confluence on six-well culture dishes were transfected with 2 μg of synthesized plasmid in 4 μL Lipofectamine2000 (Thermo Fischer Scientific, Waltham, MA, USA). After incubation for 24 h, the cells were harvested.

### 4.5. Protein Sample Preparation

Cultured cells were lysed in RIPA buffer (Cell Signaling Technology Inc., Danvers, MA, USA) containing protease inhibitor and sonicated. Excised mouse or human skeletal muscle tissue samples were disrupted by grinding for 30 s at 2000 rpm twice with a multi-bead Shocker (Yasui Kikai Co. Ltd., Osaka, Japan) in T-PER Tissue Protein Extraction Reagent (Thermo Fischer Scientifi) containing protease inhibitor. After incubation on ice for 20 min, cell lysates and tissue homogenates were centrifuged at 12,000× *g* for 20 min to remove insoluble material. The protein concentrations of the cell lysates and tissue homogenates were determined using BCA kits (Thermo Fischer Scientific). For capillary Western blotting, cell lysates were diluted to 0.01 mg protein/mL RIPA buffer, and tissue homogenates were diluted to 1 mg protein/mL T-PER Tissue Protein Extraction Reagent.

### 4.6. Simple Western Analysis

Samples were prepared and analyzed according to the manufacturer’s instructions (Protein Simple, San Jose, CA, USA). Briefly, four volumes of sample were mixed with one volume of fluorescent 5× Master Mix containing 200 mM dithiothreitol (Protein Simple) and denatured at 95 °C for 5 min. Primary antibodies against dystrophin C-terminal (ab15277; Abcam, Cambridge, MA, USA), dystrophin rod-domain (MANDYS106; EMD Millipore, Temecula, CA, USA, MANEX58; Wolfson Centre for Inherited Neuromuscular Disease, Oswestry, UK), β-actin (#4970; Cell Signaling), and vinculin (#4650S; Cell Signaling) were diluted 1:50 in antibody diluent 2 (Protein Simple). The prepared samples, the biotinylated ladder, the primary antibodies, the secondary antibodies (supplied by the manufacturer), the chemiluminescent substrate, the Stacking Matrix 2 and the Separation Matrix 2 (12–230 kDa) were dispensed into designated wells in a 384-well assay plate. The prepared assay plate was placed into the Simple Western machine (Sally Sue; Protein Simple), followed by the addition of the Simple Western assay buffers into the system tray and the insertion of capillaries. The injection volume of each sample was 40 nL. All subsequent separation, immunodetection and analysis steps were performed automatically by the machine.

Compass software (Atlanta, GA, USA) was used to visualize the Simple Western lanes, to automatically analyze signal peaks, and to calculate the area under the curve (AUC) of each peak. The ratio of Dp71 to β-actin or vinculin was calculated as the AUC of the Dp71 peak divided by the AUC of the β-actin or vinculin peak.

### 4.7. Antisense Oligonucleotide to Induce Exon 75 Skipping

An AO binding to the exonic splicing enhancer within exon 75 (20mer AODys75-3: 5′-uTuaTguTcgTgcTgcTgCu-3′, upper letters: 2′-*O*,4′-*C*-ethylene-bridged nucleic acid (ENA); lower letters: 2′-*O*-methyl RNA) was selected as described [30]. Its ability to induce exon 75 skipping was examined by transfecting this AO into adherent CRL-2061cells. Briefly, the AO was dissolved in 200 μL OptiMEM (Thermo Fishcer Scientific) and incubated for 5 min at ambient temperature. The solution was mixed with 4 μL Lipofectamine 2000 (Invitrogen) in 200 μL OptiMEM and incubated for 20 min. The mixture was added to cells in 800 μL OptiMEM, such that the final AO concentration was 50 µM. After 3 h of incubation, FBS (Gibco/Life Technologies) was added to a final concentration of 10%, and the incubation was continued for 48 h. The cells were harvested and the exon 75 covering region was RT-PCR amplified as described [46]. Experiments were done in triplicate.

### 4.8. Statistical Analysis

All statistical analyses were performed using GraphPad Prism 4.0 software (GraphPad Software Inc., la Jolla, CA, USA). All results are expressed as the mean ± S.E.M. Comparisons between two groups were performed using Mann-Whitney test, with *p* < 0.05 considered statistically significant.

## 5. Conclusions

Dystrophin Dp71 mRNA and protein were detected in human skeletal muscles using RT-PCR and the Simple Western system, respectively. Dp71 levels varied in normal human skeletal muscle samples. Dp71 was also detected in mouse skeletal muscle, showing a higher level of expression in DMD than in normal mice.
